# Supplementary material for: Evaluation of structural and ultrastructural changes in thyroid and parathyroid glands after near infrared irradiation: study on an animal model
Source: PeerJ. 2021 Aug 5;9:e11891. doi: 10.7717/peerj.11891 (PMC8349517; doi:10.7717/peerj.11891)
Supplement: Supplemental Information 2 [file peerj-09-11891-s002.pdf]

EXPOSIÇÃO A RADIAÇÃO LUMINOSA

ESPECIMEN Nº: **A** Ø D. S/Exp
IRRADIADO: Sim/ Não
DIAS PÓS-IRRADIAÇÃO: 0 dias/ 30 dias/ 60 dias.
GLÂNDULA TIROIDEIA: **Identificada/** Não identificada.
Sem alterações morfológicas/ Com alterações
GLÂNDULAS PARATIROIDEIAS: **Identificada/** Não identificadas.
Sem alterações morfológicas/ Com alterações
OBSERVAÇÕES:

EXPOSIÇÃO A RADIAÇÃO LUMINOSA

ESPECIMEN Nº: **B**
IRRADIADO: Sim/ Não
DIAS PÓS-IRRADIAÇÃO: 0 dias/ 30 dias/ 60 dias.
GLÂNDULA TIROIDEIA: **Identificada/** Não identificada.
Sem alterações morfológicas/ Com alterações
GLÂNDULAS PARATIROIDEIAS: Identificada/ **Não identificadas.** Ø D. S/Exp
Sem alterações morfológicas/ Com alterações
OBSERVAÇÕES:

EXPOSIÇÃO A RADIAÇÃO LUMINOSA

ESPECIMEN Nº: **C**
IRRADIADO: Sim/ Não
DIAS PÓS-IRRADIAÇÃO: 0 dias/ 30 dias/ 60 dias. Ø D. S/Exp
GLÂNDULA TIROIDEIA: **Identificada/** Não identificada.
Sem alterações morfológicas/ Com alterações
GLÂNDULAS PARATIROIDEIAS: **Identificada/** Não identificadas.
Sem alterações morfológicas/ Com alterações
OBSERVAÇÕES:

EXPOSIÇÃO A RADIAÇÃO LUMINOSA

ESPECIMEN Nº: **D**
IRRADIADO: Sim/ Não
DIAS PÓS-IRRADIAÇÃO: 0 dias/ 30 dias/ 60 dias. Ø D. S/Exp
GLÂNDULA TIROIDEIA: **Identificada/** Não identificada.
Sem alterações morfológicas/ Com alterações
GLÂNDULAS PARATIROIDEIAS: **Identificada/** Não identificadas.
Sem alterações morfológicas/ Com alterações
OBSERVAÇÕES:

Dra. Sara Turpin

Patologista

Sara Turpin

centro de diagnóstico anátomo-patológico, lda.
rua manuel marques, 12 c · edifício bordéus
parque europa · 1750 - 171 lisboa
tel. 217 586 940 - fax 217 586 931
e-mail: anapat@sapo.pt

EXPOSIÇÃO A RADIAÇÃO LUMINOSA

ESPECIMEN Nº: 421
IRRADIADO: Sim/ Não
DIAS PÓS-IRRADIAÇÃO: 0 dias/ 30 dias/ 60 dias.
GLÂNDULA TIROIDEIA: Identificada/ Não identificada.
Sem alterações morfológicas/ Com alterações
GLÂNDULAS PARATIROIDEIAS: Identificada/ Não identificadas.
Sem alterações morfológicas/ Com alterações
OBSERVAÇÕES:

30 D.
C/ Exp

EXPOSIÇÃO A RADIAÇÃO LUMINOSA

ESPECIMEN Nº: 422
IRRADIADO: Sim/ Não
DIAS PÓS-IRRADIAÇÃO: 0 dias/ 30 dias/ 60 dias.
GLÂNDULA TIROIDEIA: Identificada/ Não identificada.
Sem alterações morfológicas/ Com alterações
GLÂNDULAS PARATIROIDEIAS: Identificada/ Não identificadas.
Sem alterações morfológicas/ Com alterações
OBSERVAÇÕES:

30 D. C/ Exp

EXPOSIÇÃO A RADIAÇÃO LUMINOSA

ESPECIMEN Nº: 424
IRRADIADO: Sim/ Não
DIAS PÓS-IRRADIAÇÃO: 0 dias/ 30 dias/ 60 dias.
GLÂNDULA TIROIDEIA: Identificada/ Não identificada.
Sem alterações morfológicas/ Com alterações
GLÂNDULAS PARATIROIDEIAS: Identificada/ Não identificadas.
Sem alterações morfológicas/ Com alterações
OBSERVAÇÕES:

30 D.
C/ Exp

EXPOSIÇÃO A RADIAÇÃO LUMINOSA

ESPECIMEN Nº: 430
IRRADIADO: Sim/ Não
DIAS PÓS-IRRADIAÇÃO: 0 dias/ 30 dias/ 60 dias.
GLÂNDULA TIROIDEIA: Identificada/ Não identificada.
Sem alterações morfológicas/ Com alterações
GLÂNDULAS PARATIROIDEIAS: Identificada/ Não identificadas.
Sem alterações morfológicas/ Com alterações
OBSERVAÇÕES:

30 D.
S/ Exp

Dra. Paula Guerra

Patologista

centro de diagnóstico anátomo-patológico, lda.
rua manuel marques, 12 c · edifício bordéus
parque europa · 1750 - 171 lisboa
tel. 217 586 940 - fax 217 586 931
e-mail: anapat@sapo.pt

EXPOSIÇÃO A RADIAÇÃO LUMINOSA

ESPECIMEN Nº: 431 30 D. S/ 8x8
IRRADIADO: Sim/ Não
DIAS PÓS-IRRADIAÇÃO: 0 dias/ 30 dias/ 60 dias.
GLÂNDULA TIROIDEIA: Identificada/ Não identificada.
Sem alterações morfológicas/ Com alterações
GLÂNDULAS PARATIROIDEIAS: Identificada/ Não identificadas.
Sem alterações morfológicas/ Com alterações
OBSERVAÇÕES:

EXPOSIÇÃO A RADIAÇÃO LUMINOSA

ESPECIMEN Nº: 432 30 D. S/ 8x8
IRRADIADO: Sim/ Não
DIAS PÓS-IRRADIAÇÃO: 0 dias/ 30 dias/ 60 dias.
GLÂNDULA TIROIDEIA: Identificada/ Não identificada.
Sem alterações morfológicas/ Com alterações
GLÂNDULAS PARATIROIDEIAS: Identificada/ Não identificadas.
Sem alterações morfológicas/ Com alterações
OBSERVAÇÕES:

EXPOSIÇÃO A RADIAÇÃO LUMINOSA

ESPECIMEN Nº:
IRRADIADO: Sim/ Não
DIAS PÓS-IRRADIAÇÃO: 0 dias/ 30 dias/ 60 dias.
GLÂNDULA TIROIDEIA: Identificada/ Não identificada.
Sem alterações morfológicas/ Com alterações
GLÂNDULAS PARATIROIDEIAS: Identificada/ Não identificadas.
Sem alterações morfológicas/ Com alterações
OBSERVAÇÕES:

EXPOSIÇÃO A RADIAÇÃO LUMINOSA

ESPECIMEN Nº:
IRRADIADO: Sim/ Não
DIAS PÓS-IRRADIAÇÃO: 0 dias/ 30 dias/ 60 dias.
GLÂNDULA TIROIDEIA: Identificada/ Não identificada.
Sem alterações morfológicas/ Com alterações
GLÂNDULAS PARATIROIDEIAS: Identificada/ Não identificadas.
Sem alterações morfológicas/ Com alterações
OBSERVAÇÕES:

Dra. Paula Guerra

Patologista

centro de diagnóstico anátomo-patológico, lda.
rua manuel marques, 12 c · edifício bordéus
parque europa · 1750 - 171 lisboa
tel. 217 586 940 - fax 217 586 931
e-mail: anapat@sapo.pt

EXPOSIÇÃO A RADIAÇÃO LUMINOSA

ESPECIMEN Nº: 403
IRRADIADO: Sim/ Não
DIAS PÓS-IRRADIAÇÃO: 0 dias/ 30 dias/ 60 dias.
GLÂNDULA TIROIDEIA: Identificada/ Não identificada.
Sem alterações morfológicas/ Com alterações
GLÂNDULAS PARATIROIDEIAS: Identificada/ Não identificadas.
Sem alterações morfológicas/ Com alterações
OBSERVAÇÕES:

C/ 8x8
60 D

EXPOSIÇÃO A RADIAÇÃO LUMINOSA

ESPECIMEN Nº: 404
IRRADIADO: Sim/ Não
DIAS PÓS-IRRADIAÇÃO: 0 dias/ 30 dias/ 60 dias.
GLÂNDULA TIROIDEIA: Identificada/ Não identificada.
Sem alterações morfológicas/ Com alterações
GLÂNDULAS PARATIROIDEIAS: Identificada/ Não identificadas.
Sem alterações morfológicas/ Com alterações
OBSERVAÇÕES:

60 D
C/ EXP.

EXPOSIÇÃO A RADIAÇÃO LUMINOSA

ESPECIMEN Nº: 405
IRRADIADO: Sim/ Não
DIAS PÓS-IRRADIAÇÃO: 0 dias/ 30 dias/ 60 dias.
GLÂNDULA TIROIDEIA: Identificada/ Não identificada.
Sem alterações morfológicas/ Com alterações
GLÂNDULAS PARATIROIDEIAS: Identificada/ Não identificadas.
Sem alterações morfológicas/ Com alterações
OBSERVAÇÕES:

60 D
C/ EXP

EXPOSIÇÃO A RADIAÇÃO LUMINOSA

ESPECIMEN Nº: 406
IRRADIADO: Sim/ Não
DIAS PÓS-IRRADIAÇÃO: 0 dias/ 30 dias/ 60 dias.
GLÂNDULA TIROIDEIA: Identificada/ Não identificada.
Sem alterações morfológicas/ Com alterações
GLÂNDULAS PARATIROIDEIAS: Identificada/ Não identificadas.
Sem alterações morfológicas/ Com alterações
OBSERVAÇÕES:

60 D
C/ EXP.

Dra. Sara Turpin

Patologista

Sara Turpin

centro de diagnóstico anátomo-patológico, lda.
rua manuel marques, 12 c · edifício bordéus
parque europa · 1750 - 171 lisboa
tel. 217 586 940 - fax 217 586 931
e-mail: anapat@sapo.pt

EXPOSIÇÃO A RADIAÇÃO LUMINOSA

ESPECIMEN Nº: **407**
IRRADIADO: Sim/ Não
DIAS PÓS-IRRADIAÇÃO: 0 dias/ 30 dias/ 60 dias.
GLÂNDULA TIROIDEIA: **Identificada/** Não identificada.
Sem alterações morfológicas/ Com alterações
GLÂNDULAS PARATIROIDEIAS: **Identificada/** Não identificadas.
Sem alterações morfológicas/ Com alterações
OBSERVAÇÕES:

60 D 4 Exp

EXPOSIÇÃO A RADIAÇÃO LUMINOSA

ESPECIMEN Nº: **415**
IRRADIADO: Sim/ Não
DIAS PÓS-IRRADIAÇÃO: 0 dias/ 30 dias/ 60 dias.
GLÂNDULA TIROIDEIA: **Identificada/** Não identificada.
Sem alterações morfológicas/ Com alterações
GLÂNDULAS PARATIROIDEIAS: **Identificada/** Não identificadas.
Sem alterações morfológicas/ Com alterações
OBSERVAÇÕES:

60 D.
5/ Exp.

EXPOSIÇÃO A RADIAÇÃO LUMINOSA

ESPECIMEN Nº: **420**
IRRADIADO: Sim/ Não
DIAS PÓS-IRRADIAÇÃO: 0 dias/ 30 dias/ 60 dias.
GLÂNDULA TIROIDEIA: **Identificada/** Não identificada.
Sem alterações morfológicas/ Com alterações
GLÂNDULAS PARATIROIDEIAS: **Identificada/** Não identificadas.
Sem alterações morfológicas/ Com alterações
OBSERVAÇÕES:

30 D
[illegible] Exp

EXPOSIÇÃO A RADIAÇÃO LUMINOSA

ESPECIMEN Nº: **423**
IRRADIADO: Sim/ Não
DIAS PÓS-IRRADIAÇÃO: 0 dias/ 30 dias/ 60 dias.
GLÂNDULA TIROIDEIA: **Identificada/** Não identificada.
Sem alterações morfológicas/ Com alterações
GLÂNDULAS PARATIROIDEIAS: **Identificada/** Não identificadas.
Sem alterações morfológicas/ **Com alterações**
OBSERVAÇÕES: ADENOMA DA PARATIROIDEIA

30 D.
4 Exp

Dra. Sara Turpin

Patologista

Sara Turpin

centro de diagnóstico anátomo-patológico, lda.
rua manuel marques, 12 c · edifício bordéus
parque europa · 1750 - 171 lisboa
tel. 217 586 940 - fax 217 586 931
e-mail: anapat@sapo.pt

EXPOSIÇÃO A RADIAÇÃO LUMINOSA

ESPECIMEN Nº: 416
IRRADIADO: Sim/ Não
DIAS PÓS-IRRADIAÇÃO: 0 dias/ 30 dias/ 60 dias.
GLÂNDULA TIROIDEIA: Identificada/ Não identificada.
Sem alterações morfológicas/ Com alterações
GLÂNDULAS PARATIROIDEIAS: Identificada/ Não identificadas.
Sem alterações morfológicas/ Com alterações
OBSERVAÇÕES:

60 D. S/ Exp

~~60 D S/ Exp~~

EXPOSIÇÃO A RADIAÇÃO LUMINOSA

ESPECIMEN Nº: 417
IRRADIADO: Sim/ Não
DIAS PÓS-IRRADIAÇÃO: 0 dias/ 30 dias/ 60 dias.
GLÂNDULA TIROIDEIA: Identificada/ Não identificada.
Sem alterações morfológicas/ Com alterações
GLÂNDULAS PARATIROIDEIAS: Identificada/ Não identificadas.
Sem alterações morfológicas/ Com alterações
OBSERVAÇÕES:

60 D. S/ Exp.

EXPOSIÇÃO A RADIAÇÃO LUMINOSA

ESPECIMEN Nº: 418
IRRADIADO: Sim/ Não
DIAS PÓS-IRRADIAÇÃO: 0 dias/ 30 dias/ 60 dias.
GLÂNDULA TIROIDEIA: Identificada/ Não identificada.
Sem alterações morfológicas/ Com alterações
GLÂNDULAS PARATIROIDEIAS: Identificada/ Não identificadas.
Sem alterações morfológicas/ Com alterações
OBSERVAÇÕES:

30 D. C/ Exp.

EXPOSIÇÃO A RADIAÇÃO LUMINOSA

ESPECIMEN Nº: 419
IRRADIADO: Sim/ Não
DIAS PÓS-IRRADIAÇÃO: 0 dias/ 30 dias/ 60 dias.
GLÂNDULA TIROIDEIA: Identificada/ Não identificada.
Sem alterações morfológicas/ Com alterações
GLÂNDULAS PARATIROIDEIAS: Identificada/ Não identificadas.
Sem alterações morfológicas/ Com alterações
OBSERVAÇÕES:

30 D. C/ Exp

Dra. Paula Guerra

Patologista

centro de diagnóstico anátomo-patológico, lda.
rua manuel marques, 12 c · edifício bordéus
parque europa · 1750 - 171 lisboa
tel. 217 586 940 - fax 217 586 931
e-mail: anapat@sapo.pt
